# Occurrence and human risk assessment of pharmaceutically active compounds (PhACs) in indoor dust from homes, schools and offices

**DOI:** 10.1007/s11356-024-34459-4

**Published:** 2024-07-30

**Authors:** Silvia Royano, Irene Navarro, Adrián de la Torre, María Ángeles Martínez

**Affiliations:** 1 Unit of Persistent Organic Pollutants and Emerging Pollutants in the Environment, Department of Environment, CIEMAT, Avda. Complutense 40, 28040 Madrid, Spain; 2 International Doctoral School of the UNED (EIDUNED), National University of Distance Education (UNED), Madrid, Spain

**Keywords:** Pharmaceuticals, Emerging pollutants, Indoor dust, Indoor environment, UHPLC-MS/MS, Human exposure

## Abstract

This study investigates the current situation and possible health risks due to pharmaceutically active compounds (PhACs) including analgesics, antibiotics, antifungals, anti-inflammatories, psychiatric and cardiovascular drugs, and metabolites, in indoor environments. To achieve this objective, a total of 85 dust samples were collected in 2022 from three different Spanish indoor environments: homes, classrooms, and offices. The analytical method was validated meeting SANTE/2020/12830 and SANTE/12682/2019 performance criteria. All indoor dust samples except one presented at least one PhAC. Although concentration levels ranged from < LOQ to 18 µg/g, only acetaminophen, thiabendazole, clotrimazole, and anhydroerythromycin showed quantification frequencies (Qf %) above 19% with median concentrations of 166 ng/g, 74 ng/g, 25 ng/g and 14 ng/g, respectively. The PhAC distribution between dust deposited on the floor and settled on elevated (> 0.5 m) surfaces was assessed but no significant differences (p > 0.05, Mann–Whitney U-test) were found. However, concentrations quantified at the three types of locations showed significant differences (p < 0.05, Kruskal–Wallis H-test). Homes turned out to be the indoor environment with higher pharmaceutical concentrations, especially acetaminophen (678 ng/g, median). The use of these medicines and their subsequent removal from the body were identified as the main PhAC sources in indoor dust. Relationships between occupant habits, building characteristics, and/or medicine consumption and PhAC concentrations were studied. Finally, on account of concentration differences, estimated daily intakes (EDIs) for inhalation, ingestion and dermal adsorption exposure pathways were calculated for toddlers, adolescents and adults in homes, classrooms and offices separately. Results proved that dust ingestion is the main route of exposure, contributing more than 99% in all indoor environments. Moreover, PhAC intakes for all studied groups, at occupational locations (classrooms and offices) are much lower than that obtained for homes, where hazard indexes (HIs) obtained for acetaminophen (7%—12%) and clotrimazole (4%-7%) at the worst scenario (P95) highlight the need for continuous monitoring.

**Supplementary Information:**

The online version contains supplementary material available at 10.1007/s11356-024-34459-4.

## Introduction

Indoor dust is a complex matrix of particles settled on the floor, carpets, furniture or on the surface of any object. It is a mixture of biological residues (pollen, animal or insect remains, skin and hair, moulds), fibres, ash, atmospheric particulate matter (PM) and numerous PM from soil, building materials or components of any object (Maertens et al. 2004; Melymuk et al. 2020). This heterogeneous composition depends on factors such as the weather conditions, surroundings, or habits and activities developed in that environment and reflects the consumption patterns of several products and substances. For this reason, and since indoor dust is a sink for many organic pollutants, it has been widely used for the identification of emerging contaminants. Previous studies have reported the presence of numerous pollutants of emerging concern in indoor dust including perfluorinated substances (De la Torre et al. 2019), plasticizers and halogenated flame retardants (Pawar et al. 2017; Chen et al. 2019; Rantakokko et al. 2019; De la Torre et al. 2020), microplastics (Saygin, H. et al. 2023), UV filters (Ao et al. 2017), cyclic and linear siloxanes (Lu et al. 2010) or synthetic musk used for fragrances manufacture (Lu et al. 2011). However, to the author's knowledge, only three previous studies conducted in Asia have investigated the occurrence of pharmaceutically active compounds (PhACs) in indoor dust (Hoang et al. 2022; Yang et al. 2022; Lan et al. 2023). Hoang et al. 2022 found acetaminophen in 80% of samples collected from homes in the city of Hanoi (Vietnam) with a mean concentration of 295 ng/g (n = 10). Yang et al. 2022 also found high concentrations of some pharmaceutical products in Malaysia, with acetaminophen being one of the most frequently detected (Df > 80%; 51.3 ng/g median; n = 52). Finally, Lan et al. 2023 evaluated the presence of 19 anthelmintics, finding thiabendazole in domestic indoor dust samples (n = 147) in China with values ranging from 0.05 to 1078 ng/g (3.45 ng/g, median) in the dust.

The improvements in quality of life and life expectancy in recent decades are mostly attributable to the development and consumption of PhACs capable to treat chronic diseases and prevent illnesses and age-related complications (Jelic et al. 2009, OECD 2021). The occurrence of any of these compounds in the dust is of great concern since modern life forces people to spend an average of 90% of their time in indoor environments (Schweizer et al. 2007; Chen et al. 2020; Yang et al. 2022, Li and Chen 2023), and it may be a significant source of human exposure (Hwang et al. 2008; Ao et al. 2017) to these contaminants via inhalation, ingestion and dermal contact (Cao et al. 2019; Chen et al. 2019; Rantakokko et al. 2019). In this sense, studies have shown that children and especially toddlers are more vulnerable to dust-borne contaminants, since their activity pattern puts them in contact with dust, either through hand contact followed by hand-to-mouth contact or direct contact of objects with the mouth (e.g., mouthing of toys that have contacted floor or carpet dust) (Özkaynak et al. 2022). Furthermore, pollution exposure is especially dangerous at this age, since the presence of PhACs in indoor dust can affect developmental processes and trigger health problems such as endocrine disruption (Płotka-Wasylka et al. 2023).

Of all PhACs available in developed countries, consumption of antihypertensives, lipid-modifying agents, anti-diabetics and antidepressants stands out (OECD 2021). Specifically in Spain, in the last 10 years, analgesics have been the best-selling drugs, closely followed by antihypertensives such agents acting on the renin-angiotensin system and beta-blocking agents, lipid-modifying agents, anti-inflammatories, psychoanaleptics such antidepressants, antibiotics and antiepileptics (Ministry of Health 2023). After use and consumption, all these PhACs can be expelled from the body through hair or other tissues and migrate to dust particles (Saito et al. 2008). Some of these active ingredients are not only used in human pharmaceuticals but also in veterinary medicines (i.e. livestock industry or pets). Moreover, some antifungals such as thiabendazole, are also employed in agricultural applications and in food preservation to slow down putridity caused by fungi (Sun et al. 2018).

Considering all mentioned above, the present study aims to: i) obtain a reliable picture of the presence of PhACs in indoor dust from homes, classrooms and offices, ii) identify their main emission sources and iii) address the health risks posed by exposure to these contaminants for toddlers, adolescents and adults. To achieve these objectives, 22 PhACs and one metabolite were assessed in dust samples from homes, offices, and school classrooms from kindergartens and high schools in Spain. Differences in PhAC distribution between deposited and suspended dust were investigated. Data obtained were evaluated for source identification and finally, quantified concentrations were used to calculate human exposure through dust inhalation, ingestion and dermal absorption at central and worst case scenarios. To the authors’ knowledge, this is the first study that investigates pharmaceutical pollution in indoor dust from schools and offices.

## Material and methods

### Selection of target compounds

In the framework of the CEMEF project, a strengths, weaknesses, opportunities, and threats (SWOT) analysis was conducted as described elsewhere (CEMEF 2023; Royano et al. 2023). The output of the SWOT analysis was a list of emerging pollutants that included 22 PhACs containing analgesics, antibiotics, anti-inflammatories, antihypertensives, psychiatric drugs, lipid regulators, antifungals and anthelmintics. Then anhydroerythromycin, a non-active erythromycin metabolite, was added to the list. See Supplementary Material (SM) for further details about target analytes (Table S1).

### Sample collection

Eighty-five indoor dust samples were obtained from homes (n = 14), offices (n = 23), and classrooms (n = 48), from kindergarten (n = 21), and high school classrooms (n = 27) in 2022. Household samples were collected by the occupants who were asked to vacuum the entire home with their vacuum cleaners. This sampling approach provides an integrated measure of contamination and potential exposure from all rooms in which it is deployed (Harrad et al. 2010). However, the offices and classrooms sampling was carried out by the research team using a standardized protocol and equipment consisting of a paper filter placed in the vacuum cleaner tube, as shown in Figure S1. In this case, dust deposited on the floor (deposited dust), and settled on elevated (> 0.5 m) surfaces (suspended dust) were sampled. During sampling participants and the research team involved in the investigation filled in a questionnaire regarding building characteristics, outdoor surrounding characteristics and/or occupant habits. (Table S2) that were used to investigate potential contaminant sources. Nevertheless, some of this information could not be obtained for confidential reasons (schools) and representativeness (offices with more than 10 people working in shifts). Only for two offices (n = 4) with less than 10 employees, the questionnaire was filled out. Upon arrival at the laboratory, bulk dust samples were sieved through a stainless steel sieve (500 μm), homogenized, and stored at − 20 °C until analysis.

### Chemical analysis and Instrumental determination

The following method was validated for the determination of target analytes. One g of indoor dust spiked with 50 ng of deuterated standards (acetaminophen-d3, atenolol-d7, gemfibrozil-d6, ibuprofen-d3, sulfamethoxazole-d4, and venlafaxine-d6) was extracted with a mixture of 2 mL of Milli-Q water and 4 mL of acetonitrile acidified with 1% acetic acid and stirred for 30 min in a mechanical shaker. Then, 0.8 g magnesium sulfate and 0.2 g sodium acetate were added. The mixture was vortexed for 30 s, centrifuged for 5 min at 5000 rpm and the supernatant was evaporated to 1 mL and spiked with 10 ng of clothianidin-d3. Finally, 500 µL of the extract was filtered (0.45 µm PTFE vial filter) prior to instrumental analysis. Chromatographic separation was conducted with an ultra high performance liquid chromatograph (UHPLC) ExionLC system (SCIEX, MA) using a 1.6 µm C18 100 Å column (100 × 2.1 mm i.d, Phenomenex). UHPLC system was coupled to a Triple Quad™ 3500 MS/MS System (SCIEX, MA) equipped with a Turbo V™ ion source (SCIEX, MA). Details related to PhAC instrumental determination have been previously published (Royano et al. 2023) and are summarized in SM.

### QA/QC and statistical analysis

The validated method fulfilled SANTE/2020/12830 (European Commission 2020) and SANTE/12682/2019 (European Commission 2019) performance criteria. The detailed results of validation experiments are compiled in SM (Table S5). The lowest validated concentration level meeting criteria for recovery (70—120%), precision (RSD ≤ 20%), and identification (MS/MS ion ratio within 30%), was established as the limit of quantification (LOQ). LOQs were 5 ng/g for all PhACs except acetaminophen, and atorvastatin (50 ng/g). Unfortunately, the method could not be validated for ibuprofen and ketoprofen since these analytes did not fulfil SANTE recovery criteria. Limits of detection (LODs), as the concentration giving a signal to noise ratio of 3:1 were calculated from the qualifying transition of the lowest validated level (LOQ), ranged from 1 to 16 ng/g. Procedural blanks (diatomaceous earth vacuumed with the same vacuum cleaner used for sampling) were processed and analyzed as samples. Furthermore, acetonitrile injections were run between sample injections as instrumental blanks to check carryover and contamination from the chromatograph system. All PhACs were below LODs in procedural and instrumental blanks and recoveries of surrogate standards ranged between 72—94%.

SPSS 14.0 for Windows was used to perform the statistical analyses. For the descriptive statistical analysis, PhAC concentrations below LOQs but with total quantification frequencies (Qfs) above 30%, were replaced by LOQs divided by the square root of 2. Analyte concentrations were not normally distributed (p < 0.05, Shapiro–Wilk W and Kolmogorov–Smirnov tests). Therefore, to investigate bivariate relationships Spearman rank correlation coefficient was derived but in this case, only values > LOQs were included. Kruskal–Wallis H and Mann–Whitney U tests were run to evaluate statistical differences between indoor environments as well as deposited and suspended dust distributions. Wilcoxon test was run to assess statistical differences between PhACs in the same indoor environments.

### Calculation of risk assessment parameters

Human exposure to indoor dust can mainly occur through inhalation of suspended dust, ingestion of deposited dust, and dermal absorption of both matrices. Thus, estimated daily intakes (EDIs) were calculated for exposure at central (P50; median) and worst (P95) scenarios according to Eq. 1, 2, and 3 (U.S. EPA 2011, De la Torre et al. 2020, and Zhu et al. 2023) for adults (20 to 60 years), adolescents (10 to 17 years) and toddlers (1 to 3 years) (EFSA 2011) separately.

1 $$ED{I}_{inhalation}=\frac{{C}_{dust}\; x\; I{R}_{inhalation} \; x\; F}{BW \; x\; PEF}$$

2 $$ED{I}_{ingestion}=\frac{{C}_{dust} \; x\; IngR \; x\; F}{BW}$$

3 $$ED{I}_{dermal}=\frac{{C}_{dust}\; x\; DAS\; x\; ESA\; x\; A{F}_{dermal}\; x\; F }{BW}$$

C_dust_ is the analyte concentration (P50 and P95; ng/g). IR_inhalation_ is the inhalation rate (m^3^/day). F stands for the fraction of time spent in the specific exposure situation (homes, schools and offices) in a day (unitless). BW is the average body weight (14 kg for toddlers, 57 kg for adolescents and 80 kg for adults; U.S. EPA 2011). PEF is the particle emission factor (m^3^/g). IngR describes the ingestion rate of indoor dust (g/day). DAS is the dust adhered to skin rate (mg/cm^2^). ESA is the exposed skin area (cm^2^/day). AF_dermal_ is the dermal absorption fraction (unitless). Complete details of data used for EDI calculations are compiled in Table S6.

EDIs were used to calculate hazard quotient (HQ) as the ratio of the chronic daily intake (CDI; ng/kg/day) to the chronic reference dose (RfD; ng/kg/day) (Duong et al. 2023), where EF is the exposure frequency (day/year), ED is the exposure duration (years) and AT is the average lifetime (days).

4 $$HQ=\frac{CD{I}_{inhalation}\; or\; CD{I}_{ingestion} \; or\; CD{I}_{dermal}}{RfD}\;x\;100=\frac{\frac{EDI\; x\; EF\; x\; ED}{AT}}{RfD }\;x\;100$$

Finally, the hazard index (HI) was obtained as the sum of HQs calculated for inhalation, ingestion and dermal exposure, to assess the potential risk of adverse health effects from the three exposure routes. If HI is below 100% no adverse health effects on humans can be considered. In contrast, if HI is above 100%, the negative impacts on human health are considered (Duong et al. 2023; Sun et al. 2023).

5 $$HI=\sum H{Q}_{i}$$

## Results and discussion

### PhAC distribution in indoor dust

Main descriptive statistics for households, classrooms and offices were calculated and summarized in Table S7. It is worth mentioning that indoor dust samples are a complex matrix, and therefore the results showed a wide variability. However, no statistically significant (p > 0.05, Mann–Whitney U-test) differences were obtained between floor-deposited and suspended (> 0.5 m) dust, and therefore all samples were combined and discussed together (Cequier et al. 2014). From all the target analytes, acetaminophen, thiabendazole, clotrimazole and anhydroerythromycin seemed to be the main contaminants presented in indoor dust samples with quantification frequencies higher than 19% and median concentrations of 166 ng/g, 74 ng/g, 25 ng/g and 14 ng/g, respectively. Atenolol, carbamazepine, clarithromycin, erythromycin, fluconazole, irbesartan, metoprolol, miconazole, naproxen, sulfamethoxazole, trimethoprim, and valsartan were quantified in less than 11% of the samples and their concentration ranged from 5.2 ng/g to 630 ng/g. In contrast, seven PhACs (atorvastatin, azithromycin, gemfibrozil, ketoprofen, ibuprofen, o-desmethylvenlafaxine, and venlafaxine) were below LODs in all cases. The adsorption properties of PhACs on the dust at first could be derived from adsorption coefficients on organic carbon (Koc) but are challenging to predict due to the interactions with specific functional groups or pH-dependent characteristics (Duong et al. 2023). Some studies have investigated the adsorption capacity of different PhACs on adsorbents such as synthetic polymers like chitosan, cellulose, or biochar (Zhang et al. 2016; Varadharajan et al. 2022; Petrie et al. 2023) and the PhACs sorption mecanisms on microplastics (Upadhyay et al. 2022). These studies demonstrated that charge attraction is one of the main interactions between pharmaceuticals and adsorbents (Zhang et al. 2016). Nonetheless, the heterogeneity and complexity of the dust composition could influence any expected behaviour and make any prediction difficult.

**Fig. 1:**
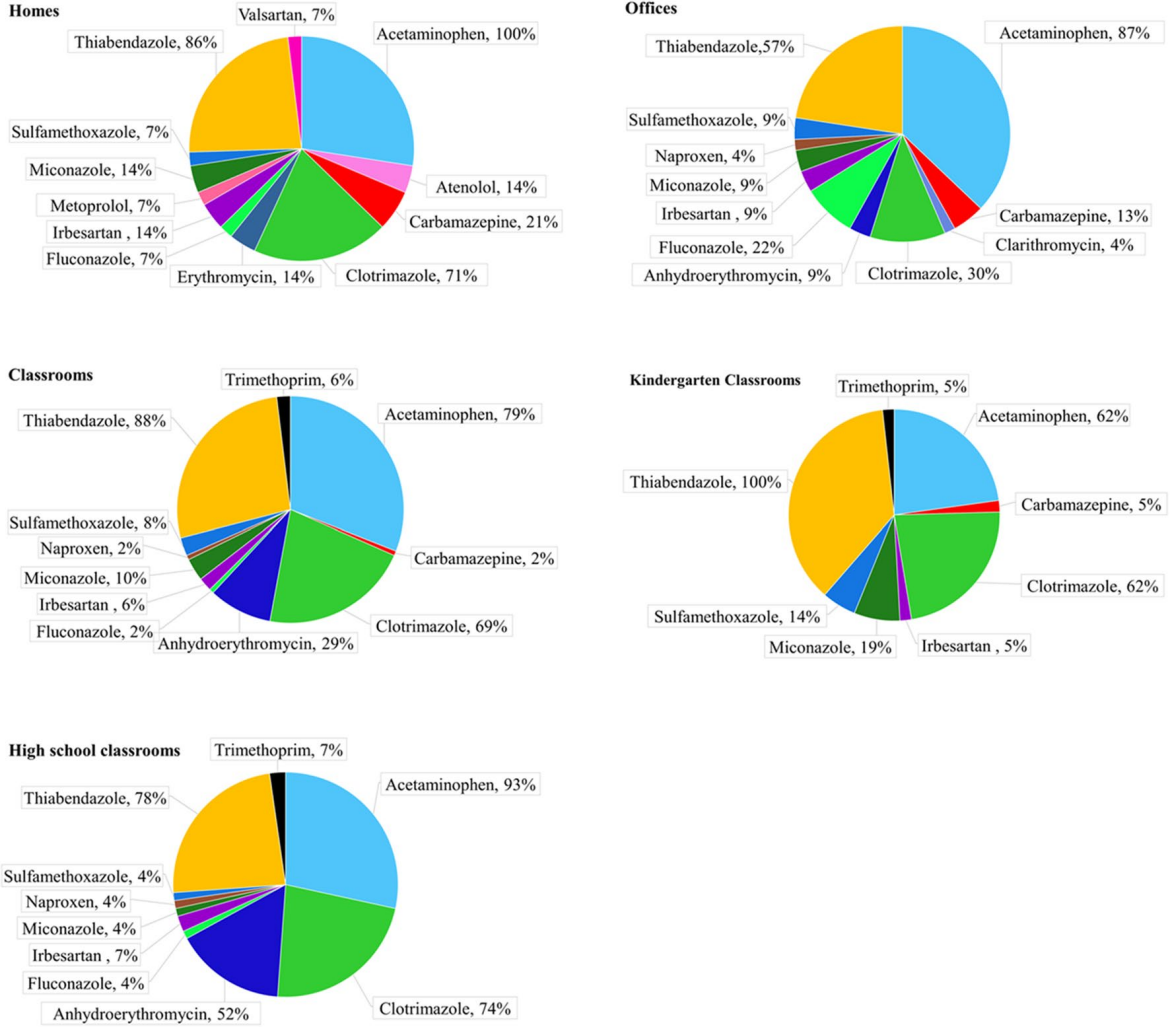
Quantification frequencies (%) of PhACs in the investigated indoor environments

PhACs were quantified in all samples but interesting differences were obtained between indoor environments (Fig. 1). Acetaminophen and thiabendazole presented Qfs above 50% in homes, classrooms and offices (Table S7). Nonetheless, in household dust, clotrimazole was also quantified in 71% of the samples (22 ng/g; median) followed by carbamazepine (21%, 66 ng/g) and to a lesser extent by atenolol (14%, 115 ng/g), erythromycin (14%, 61 ng/g), irbesartan (14%, 17 ng/g), and miconazole (14%, 10 ng/g). Anhydroerythromycin were below LOQ in all household samples. In the case of offices, acetaminophen (87%, 104 ng/g) and thiabendazole (57%, 73 ng/g) were followed by clotrimazole (30%, 15 ng/g), fluconazole (22%, 22 ng/g), and carbamazepine (13%, 51 ng/g). Considering all sampled classrooms, acetaminophen, clotrimazole, anhydroerythromycin, and thiabendazole were the most prevalent compounds (Qfs > 29%). Both types of classrooms presented a similar PhAC pattern, dominated by thiabendazole (100—78%, 79 – 72 ng/g; % and medians for kindergarten and high school classrooms, Table S8), acetaminophen (62 – 93%, 79—223 ng/g) and clotrimazole (62 – 74%, 25 –29 ng/g) and to a lesser extent by sulfamethoxazole (14 – 4%, 19 –33 ng/g), and miconazole (19 – 4%, 6.9 – 15 ng/g). However, it is noteworthy to mention that while anhydroerythromycin was below LOQ in all kindergartens, it was quantified in 52% (15 ng/g; median) of the high school classrooms.

**Fig. 2:**
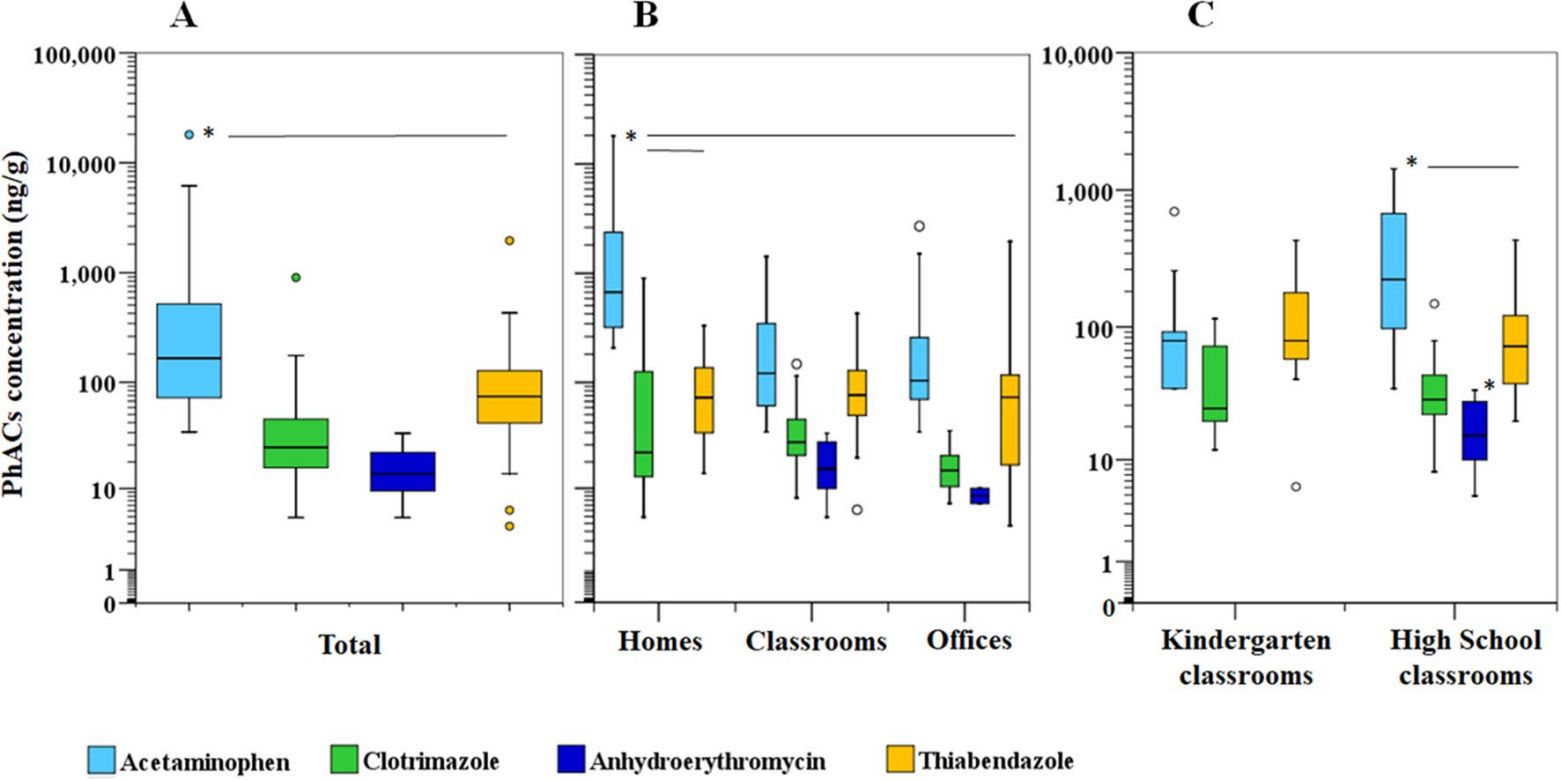
Box and whiskers plot of main quantified PhACs (concentrations; ng/g, logarithmic scale) in dust from all samples (**A**), each indoor environment evaluated (homes, classrooms and offices) (**B**), and different classroom types (kindergartens and high school classrooms) (**C**). * Asterisk denotes statistically significant (p < 0.05) differences

Acetaminophen showed significantly higher (p < 0.05, Wilcoxon test) concentrations in comparison to the other PhACs for the total of the samples (Fig. 2A), but also for homes and high school classrooms (Figs. 2B and 2C). However, acetaminophen median concentration did not show significant differences (p > 0.05, Wilcoxon test) with thiabendazole in kindergarten classrooms and offices (Fig. 2B and 2C). Moreover, acetaminophen levels found in homes were significantly higher than those obtained in classrooms and offices (Fig. 2B; p < 0.01, Kruskal–Wallis H-test). This fact is corroborated by the information gathered from questionnaires since acetaminophen was consumed in 13 of the 14 households sampled. Interestingly, the lowest median acetaminophen concentration was found in kindergarten classrooms (79 ng/g; Table S8) and this was three times lower than the one obtained in high school classrooms (223 ng/g). Furthermore, the quantification frequency in the youngest students' classrooms was 62%, representing the lowest Qf in indoor environments, compared to 93% in high school classrooms. Acetaminophen is one of the most widely used over-the-counter analgesics and antipyretics worldwide (Vo et al. 2019, Roberts et al. 2015). According to the Spanish Agency for Medicines and Health Products, 261 authorized medicines contain acetaminophen and it is the main active ingredient of 121 (AEMPS 2023). Of all PhACs analysed acetaminophen is the most soluble in water (Table S1) and therefore it is plausible to think that once consumed it will be mainly excreted in the urine. Nevertheless, acetaminophen can be incorporated into hair or nails (Saito et al. 2008) and expelled from the body in biological residues that become part of the indoor dust.

No statistically significant differences (p > 0.05, Kruskal–Wallis H-test) were obtained between households, offices and classrooms in terms of thiabendazole levels (Fig. 2B and 2C). However, in kindergarten classrooms, it was quantified at similar concentrations (79 ng/g; median Table S8) than acetaminophen (79 ng/g). Thiabendazole is a parasiticide, a fungicide and also a food additive. Therefore, any of these uses could be responsible for its presence in the dust. In Spain, 17 authorized post-harvest use pesticide formulations contain thiabendazole in accordance with the register of phytosanitary products of the Ministry of Agriculture, Fisheries and Food (Ministry of Agriculture, Fisheries and Food 2023). It is also used to prevent fungal growth on vegetables and fruits during transportation and prolonged storage (Müller et al. 2014) and is employed as a food preservative (E233). Thiabendazole is also used in the manufacture of wiping cloths and scouring pads as an antibacterial treatment to retard bacterial growth and odours. These non-pharmacological sources could be the origin for 93% of the sampled homes that reported not having this medicine. Nevertheless, in kindergarten schools, thiabendazole anthelmintic used as a treatment for diseases caused by parasites may play a more important role. These pathologies affect approximately 3.5 billion people every year and cause more than 450 million health problems worldwide (Fauziah et al. 2022) but affect the paediatric population more than adults (Fumadó, 2015).

Similar clotrimazole concentrations were found between indoor environments, and only offices presented lower (p < 0.05 Mann–Whitney U test) median concentration (15 ng/g) than classrooms (28 ng/g). There are currently 17 authorised clotrimazole-containing products in Spain mainly creams and powders for topical use, which are available on prescription and over-the-counter (AEMPS 2023) to treat vaginal yeast infections, oral thrush, diaper rash, tinea versicolor, and types of ringworm including athlete's foot and onychomycosis. Therefore, as mentioned for acetaminophen, the incorporation and elimination of clotrimazole through the hair, skin, sweat or other biological tissues would be a potential source of clotrimazole dust contamination. However, the use of this medication may not be evident in all cases since it was quantified in 5 homes where its use was not recognized.

It has been described that erythromycin as other macrolide antibiotics, degrades under acidic conditions into anhydroerythromycin and exists principally in the degraded form in aquatic environments (Wang et al. 2012). Therefore, it is not strange that anhydroerythromycin concentrations have been reported in surface waters (Royano et al. 2023) and river sediments (Li et al. 2018 and 2019). Anhydroerythromycin was quantified (14 ng/g, 5.1 and 34 ng/g; median, min – max, Table S7) in 19% of the dust samples which suggested that erythromycin degradation could also occur indoors. Nevertheless, data did not show a direct relationship between the precursor and its metabolite. In fact, erythromycin was only quantified in only 2 homes while anhydroerythromycin levels were below LOQ in all homes and kindergarten schools and only quantified in offices (9%, 8.7 n/g; Qf, median) and especially in high school classrooms (52%, 15 ng/g). This elevated Qf is probably due to the increased prescription of antibiotics, particularly macrolides, at younger ages and in adolescence (Holstiege et al. 2015). In Spain, erythromycin is the most widely used macrolide to treat skin infections and acne, which presents high incidence in adolescents (Muñoz 2001, EASP 2019) and it can be found in products such as wipes or gels for topical use, unlike other macrolides such as azithromycin and clarithromycin, which are only found in formulations for oral use (AEMPS 2023). This may explain why the presence of erythromycin and its metabolite anhydroerythromycin was more pronounced than that of other macrolides.

**Fig. 3:**
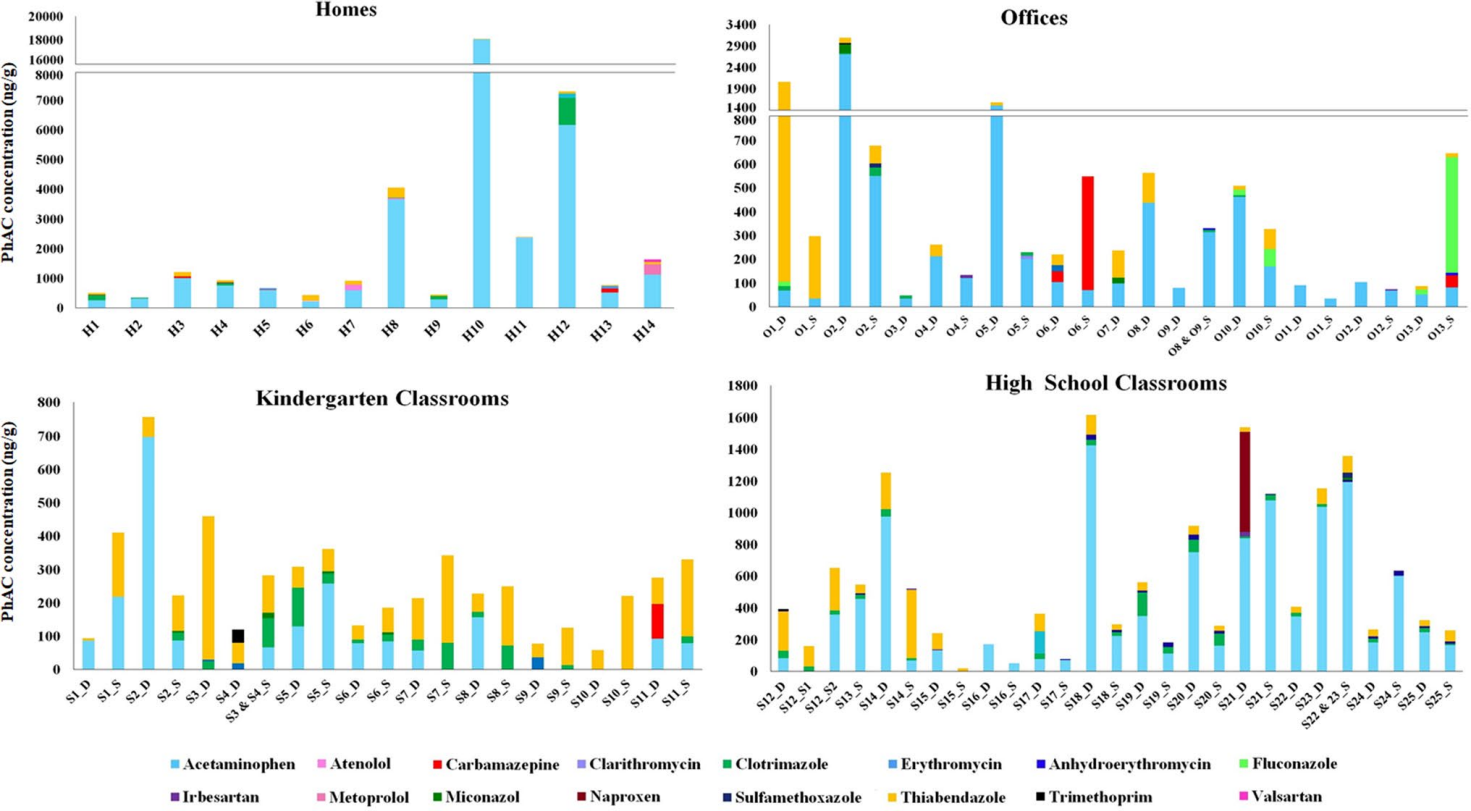
Concentrations (ng/g) for PhACs found in dust collected from homes (H), offices (O) and school classrooms (S), differentiating between kindergarten schools and high schools. Samples of suspended dust (S) and deposited dust (D) are also shown for each location if available

Atenolol, carbamazepine, clarithromycin, erythromycin, fluconazole, irbesartan, metoprolol, miconazole, naproxen, sulfamethoxazole, trimethoprim, and valsartan were quantified in at least one sample of each indoor environment (Fig. 3). The beta blocker atenolol was only quantified in two homes where its use was not reported. Similarly, the antiepileptic carbamazepine was found in three homes of which only one of them used this medicine. It was also present in one kindergarten classroom and three offices. On the other hand, the fungicide fluconazole was quantified in one home that recognized its use and was also present in one high school classroom and five offices. The antihypertensive irbesartan was quantified in two homes where inhabitants reported its use in the questionnaires. One kindergarten, two high school classrooms and two offices also presented irbesartan levels. The fungicide miconazole was found in two homes where the use of fungicide drugs was communicated. Besides, it was quantified in four kindergarten classrooms, one high school classroom and two offices. The antibiotic sulfamethoxazole was presented in one home, three kindergarten classrooms, one high school classroom and two offices. This PhAC is often used in combination with trimethoprim in relation (1:5), but as happened for erythromycin and its metabolite, no associations were found between both antibiotics. Trimethoprim was only quantified in one kindergarten and two high school classrooms. Both antibiotics have similar physical and chemical characteristics, dominated by their high solubility in water (Table S1) and therefore once administrated may be mainly excreted in the urine. Metoprolol and valsartan contamination found in home H14 correlates with the use of antihypertensives by their occupants. However, in some cases, an unequivocal use was not clearly identified. Home H13 reported no antibiotic use but presented an erythromycin concentration of 84 ng/g in the dust. After investigating its potential origin, the use of wipes containing this antibiotic to treat acne was suggested. In general, data revealed that the presence of drugs in indoor dust could be attributed to their use and excretion in sweat, hair or skin. Nevertheless, other PhAC emission sources should not be ruled out since in some cases, concentration levels above LOQ were quantified in locations where their use was not evident.

Information gathered using questionnaires was utilised to evaluate potential relationships between PhAC dust levels and occupant habits, building characteristics, and/or outdoor surroundings. PhAC concentration differences between locations used or not used antidepressants, antiepileptics, antibiotics, antihypertensives, lipid regulators, analgesics, anti-inflammatories, antifungals and anthelmintics were explored (Figure S2). Nonetheless, only homes that use fungicides presented higher (p < 0.05 Mann Whitney U test) dust concentrations compared to those that do not use them. This result highlights that pharmacological use is a more relevant source than non-pharmacological ones. At this point, it must be mentioned that household sampling was conducted according to availability and therefore in the case of analgesics, antiepileptics, and anthelmintics (Figure S2) sampling size did not allow a direct comparison. Similar behaviour was revealed in the Spearman’s Rho correlation matrix (Table S9) where significant correlations were based on less than 4 pairs of data and were discarded from the discussion.

Information about pharmaceuticals in dust samples is rather scarce. To the author's knowledge, this is the first time that drug concentrations in indoor dust were reported for schools and offices. Only three previous studies have recently described the presence of PhACs in household dust from Asia (Hoang et al. 2022, Lan et al. 2023 and Yang et al. 2022). Data reported in the present study for acetaminophen in household dust (2567, 209–18048 ng/g; median, min – max) are similar to those described by Yang et al 2022 in homes from Malaysia (8.7 to 11785 ng/g and 92% QF) but higher than those obtained by Hoang et al. 2022 (295, 38–1480 ng/g; average, min–max) in Vietnamese households. Interestingly, Yang et al. 2022 also reported metoprolol (192 ng/g; 1 sample) and sulfamethoxazole (19 ng/g; median) dust concentrations at similar levels and Qfs than those obtained in homes evaluated here (351 and 10 ng/g; Table S7).

As mentioned before thiabendazole is used as a pesticide and antifungal in food preservation, therefore, its occurrence has been described more frequently in the literature (Lan et al. 2023, Liu et al. 2022, Salis et al. 2017, Shin et al. 2020, and Yang et al. 2022) with values ranging from 0.05 to 2495 in indoor dust. Specifically, Navarro et al. 2023 recently investigated the occurrence of plant protection products in indoor dust from farmworker households across Europe, and Argentina and values described for thiabendazole (0.1 to 1275 ng/g,) are similar to those obtained here (5.0- 1960 ng/g).

### Human exposure assessment

**Fig. 4:**
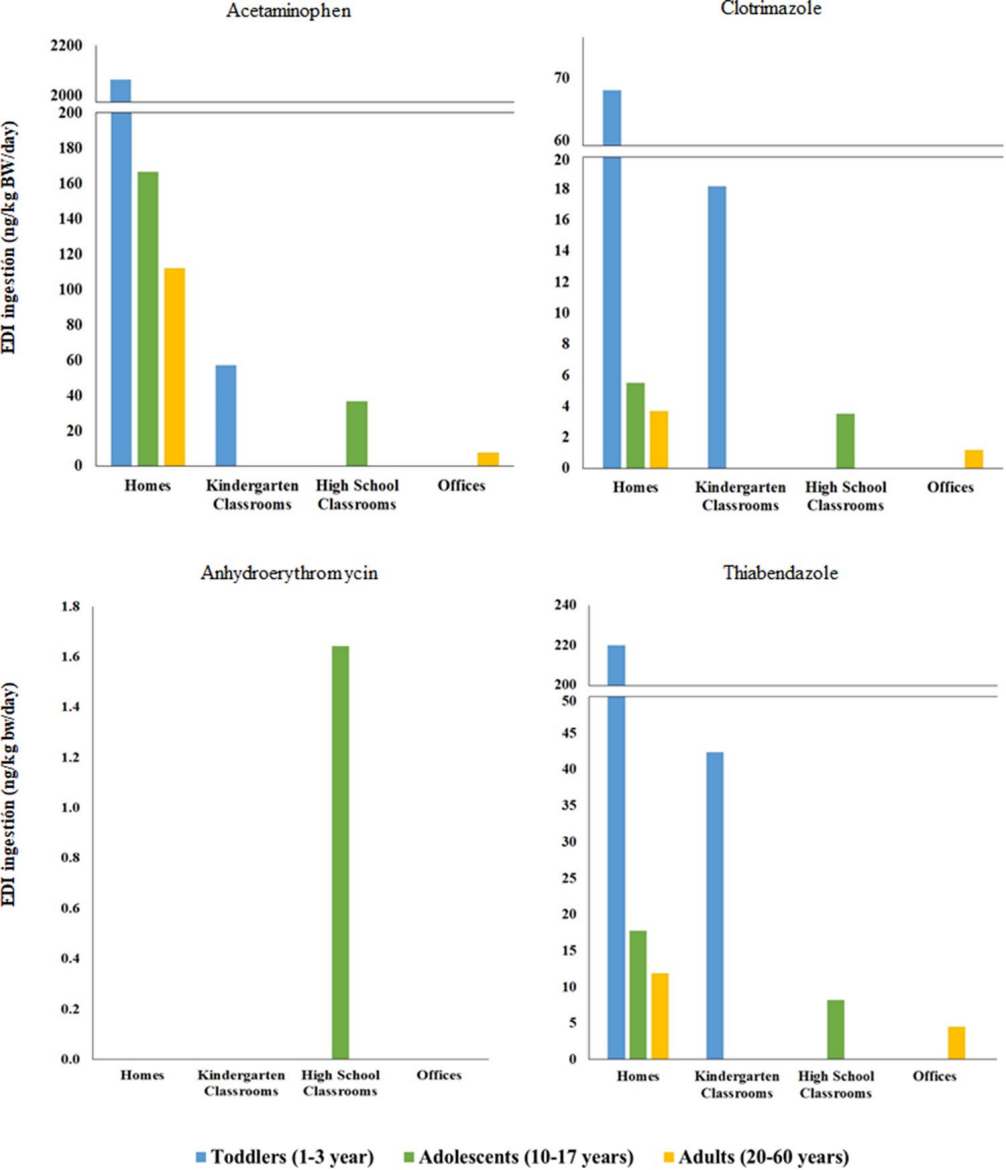
Estimated daily intake (ng/kg BW/day) calculated for the central scenario for toddlers (blue), adolescents (green) and adults (yelow) for the four most frequent PhACs at each location

There is no doubt that human exposure to PhACs is mainly due to the consumption of medicines. However, data obtained in the present study, suggest that inhalation, ingestion and/or dermal absorption of indoor polluted dust may also represent a source of human exposure to pharmaceuticals that must be investigated. As toddlers, adolescents and adults do not spend the same amount of time in the evaluated locations, the exposure duration will be different for each age group. Thus, estimated daily intakes via dust inhalation (EDI _inhalation_), ingestion (EDI _ingestion_) or dermal absorption (EDI _dermal_) were calculated for two exposure scenarios (using the P50 and P95 concentrations, respectively) and for toddlers, adolescents, and adults separately. Inhalation EDIs were calculated considering only PhAC concentrations obtained in suspended dust. In the same way, for ingestion EDIs calculation only data obtained for deposited dust samples were used. Finally, concentrations from suspended and deposited were combined to estimate dermal EDI values. As no distinction was made between suspended and deposited dust in the homes, the EDIs were calculated for the total dust collected in these locations. Moreover, since results revealed PhAC significant differences between indoor environments, EDIs and HIs were calculated for each indoor environment (Tables S10, S11, and S12). In the three indoor environments (homes, offices and classrooms), the results evidenced that dust ingestion represents a higher contribution for all age groups, compared to dermal absorption and inhalation (< 1% in homes). Therefore, discussion related to human exposure was referred only to EDI _ingestion_. As expected, EDI _ingestion_ values for toddlers were higher than for adolescents and adults. Acetaminophen was the pharmaceutical with higher EDI for toddlers in homes (2063 and 62,877 ng/kg BW/day; median and worst case scenarios) and kindergarten classrooms (57—619 ng/kg BW/day), but contribution in the former is 40–100 times higher compare to the latter. In the case of adults, acetaminophen EDI _ingestion_ value (7.8 ng/kg BW/day) was slightly surpassed by miconazole (8.7 ng/kg BW/day) in offices at the central scenario (Fig. 4), but in the worst case, acetaminophen and thiabendazole offered the highest exposure rates (448 and 255 ng/kg BW/day respectively). Acetaminophen also accounted for the highest EDI _ingestion_ in high school classrooms for adolescents at median (37 ng/kg BW/day) and worst case (377 ng/kg BW/day) but again these levels are low compared to homes revealing that, as mentioned for toddlers, PhAC intakes for adolescents and adults at occupational locations (high school classrooms and offices) are much lower than that obtained for homes. In homes, acetaminophen intake rate was followed by atenolol, thiabendazole, carbamazepine, erythromycin, clotrimazole, irbesartan, and miconazole (Table S10). In the worst scenario, clotrimazole was the second PhAC with a higher EDI value. In contrast, the intake of miconazole in the central scenario seemed to be the highest in offices followed by acetaminophen, thiabendazole, fluconazole, clotrimazole, and anhydroerythromycin, and carbamazepine (Table S10). In classrooms, acetaminophen was mainly followed by thiabendazole and clotrimazole (Table S10). As mentioned before, in most cases, the consumption of medicines is the main route of exposure to PhACs, but ingestion of indoor dust could represent a continuous exposure to drugs for people who do not need or are not able to take these medicines. Finally, EDIs were used to calculate HI according to Eq. 4. Results listed in Table S12 revealed HIs below 1% of RfD for most PhACs indicating that their presence in the dust may not have adverse effects on human health. Nevertheless, HIs values obtained for acetaminophen (7%—12%) and clotrimazole (4%—7%) for all age groups in homes at worst scenarios highlight the need for continuous monitoring. The results obtained in this work were in accordance with those reported in other studies. The values ​​obtained for EDI and HQ were of the same order of magnitude as those calculated by Hoang et al. 2022, Yang et al. 2022 and Lan et al. 2023 (ranging from 1 ng/Kg BW/day to 300 ng/Kg BW/day). Furthermore, all the works conclude in a negligible risk for human health but point out the need to continue investigating their presence in indoor environments due to the toxicity that these compounds could present.

## Conclusions

Indoor dust samples were collected from three indoor environments where people spend most of their time (homes, classrooms and offices) to investigate PhAC indoor contamination. To the authors’ knowledge, this is the first study that reported pharmaceutical pollution in indoor dust from schools and offices. Acetaminophen, clotrimazole, anhydroerythromycin and thiabendazole were the main PhACs, quantified in more than 19% of the 85 analyzed samples with median levels ranging from 166 ng/g to 14 ng/g. Acetaminophen was the PhAC with higher concentration levels in all locations. Despite its presence in work and educational environments, it was in homes that both toddlers and adults were most exposed to acetaminophen. The principal source of drugs in indoor dust could be attributed to their consumption and subsequent elimination from the body. No relationships were found between occupant habits, building characteristics, and/or outdoor surroundings and PhAC presence in dust samples. Only antifungals revealed a correlation between drug consumption and PhAC occurrence. No differences were found between suspended and deposited dust. Homes were found to be the places with the highest estimated daily intakes, being the toddler's exposure rate higher than the adults in all cases. Finally, although according to the hazard indexes obtained no adverse impacts on human health should be expected, it is strongly recommended to frequently vacuum and ventilate indoor environments, especially in places where there are toddlers.

### Supplementary Information

Below is the link to the electronic supplementary material.

Supplementary file1 (DOCX 2484 KB)

Supplementary file2 (XLSX 22 KB)

## Data Availability

The datasets used and/or analyzed during the current study are available from the corresponding author on reasonable request.
