# Supplementary material for: Occurrence and human risk assessment of pharmaceutically active compounds (PhACs) in indoor dust from homes, schools and offices
Source: Environ Sci Pollut Res Int. 2024 Jul 30;31(37):49682–93. doi: 10.1007/s11356-024-34459-4 (PMC11324665; doi:10.1007/s11356-024-34459-4)
Supplement: Supplementary file 1 — Supplementary file1 (DOCX 2484 KB) [file 11356_2024_34459_MOESM1_ESM.docx]

# SUPPLEMENTARY MATERIAL

**Occurrence and human risk assessment of pharmaceutically active compounds (PhACs) in indoor dust from homes, schools and offices.**

Silvia Royano^1,2^, Irene Navarro^1^, Adrián de la Torre^1,3*^, María Ángeles Martínez^1,3^

^1^ Unit of Persistent Organic Pollutants and Emerging Pollutants in the Environment, Department of Environment, CIEMAT, Avda. Complutense 40, 28040, Madrid, Spain.

^2^ International Doctoral School of the UNED (EIDUNED), National University of Distance Education (UNED), Madrid, Spain.

^3^ These authors share last authorship

^*^ Corresponding author: adrian.delatorre@ciemat.es

**Table of content**

|  | **Page** |
| --- | --- |
| **S1. Chemicals and materials……………………………………………………………………….** | S3 |
| **S2. Instrumental analysis** | S7 |
| **S3. Analytical method validation ………………………………………………………………….** | S9 |
| **S4. Human exposure assessment ………………………………………...………………………...** | S10 |
| **Figures** |  |
| **Figure S1.** (A) Paper filter used for offices and classrooms sampling. (B) Vacuum cleaner arrangement for suspended (>0.5m) dust sampling. (C) Vacuum cleaner arrangement for deposited (floor) dust sampling. (D) Dust sample collected. (F) Sieved (500 µm) dust sample…..... | S6 |
| **Figure S2.** Box and whiskers plot of the pharmaceutical concentration by therapeutic groups related to use and storage according to information from the questionnaires. N is the number of homes and offices where each group of PhAC has been used or not used in the last year.…………. | S13 |
| **Tables** |  |
| **Table S1.** CAS Nº, molecular weight, pKa, water solubility, lipophilicity (logKow), soil affinity (logKoc), biodegradation rates and applications for target PhACs..………………………………… | S4 |
| **Table S2.** Details of questionnaires collected related to building characteristics, inhabitant habits and PhAC consumption.…………………………..…………………………………………………. | S5 |
| **Table S3.** UHPLC-ESI conditions…..………………………………………………………………. | S7 |
| **Table S4.** Precursors and product ions (quantifier and qualifier), collision energies (CE), declustering potential (DP) and collision cell exit potential (CXP) selected for the Multiple Reaction Mode (MRM) analysis………………………………………………................................. | S8 |
| **Table S5**. Limits of detection (LODs), limits of quantification (LOQs) and validation results...…... | S9 |
| **Table S6.** Parameter used for EDIs calculation…...……………….………………………………... | S10 |
| **Table S7.** Quantification frequencies (Qf %), median (min. – max.) concentration (ng/g) obtained in dust from homes, classrooms and offices. ……………………………………………………… | S11 |
| **Table S8.** Quantification frequencies (Qf %) and median (min. – max.) concentration (ng/g) obtained in dust from kindergarten and high school classrooms.…………………………................ | S12 |
| **Table S9.** Spearman`s Rho correlation matrix. …………….................................... Excel document |  |
| **Table S10.** Estimated daily intakes (ng/kg BW/day) via dust inhalation (EDI inhalation), ingestion (EDI ingestion), and dermal absorption (EDI dermal) calculated for toddlers, adolescents, and adults at central (P50) and worst (P95) scenarios. Only PhACs that were quantified in at least two samples were considered for EDIs estimation………………………………………………………. | S14 |
| **Table S11.** Hazard Quotient (HQ) calculated for toddlers, adolescents, and adults at central (P50) and worst (P95) scenarios. (T: toddlers; Ad: adolescents; A: adults)……………………………….. | S15 |
| **Table S12.** Hazard Index (HI) calculated for toddlers, adolescents, and adults at central (P50) and worst (P95) scenarios. ………...…………………………………… | S16 |

**S1. Chemicals and materials.**

Native and deuterated standards were acquired from LGC Dr. Ehrenstorfer (Augsburg, Germany) (carbamazepine, clarithromycin, clotrimazole, erythromycin, fluconazole, ibuprofen-d3, irbesartan, naproxen, o-desmethylvenlafaxine, sulfamethoxazole, sulfamethoxazole-d4, thiabendazole, trimethoprim, and valsartan), LabStandard (Barcelona, Spain) (acetaminophen, acetaminophen-d3, atenolol, atenolol-d7, atorvastatin, azithromycin, gemfibrozil, ibuprofen, ketoprofen, metoprolol, miconazole, and venlafaxine,), Toronto Research Chem Canada (North York, ON, Canada) (gemfibrozil-d6) and LGC LoGiCal (Luckenwalde, Germany) (velafaxine-d6). Methanol (LC-MS grade) was obtained from J.T. Baker (Philipsburg, NJ, USA). Acetonitrile (HPLC grade) was purchased from SYMTA S.L.L (Madrid, Spain). Formic acid and acetic acid were obtained from Scharlau (Barcelona, Spain). Magnesium sulfate and sodium acetate were purchased from Merck KGaA (Darmstadt, Germany).

Individual stock solutions were used for preparing native and deuterated working standard mixtures in acetonitrile at a concentration of 1 mg/L. All standard solutions were stored at -20ºC.

**Table S1.** CAS Nº, molecular weight, pKa, water solubility, lipophilicity (logKow), soil affinity (logKoc), biodegradation rates and applications for target PhACs.

| Category | PhAC | CAS Nº | MW (g/mol) ^a^ | pKa ^a^ | Water  solubility  (mg/L) ^a^ | logKow ^a^ | LogKoc ^a^ | Biodegradation ^a^ | Human / Veterinary ^b^ |
| --- | --- | --- | --- | --- | --- | --- | --- | --- | --- |
| Antibiotics | Azithromycin | 83905-01-5 | 749 | 8.5 | 0.062 | 4.02 | 1.68 | Weeks-months | Yes / No |
|  | Clarithromycin | 81103-11-9 | 748 | 9.0 | 0.342 | 3.16 | 1.37 | Weeks-months | Yes / No |
|  | Erythromycin | 114-07-8 | 734 | 8.9 | 0.517 | 3.06 | 1.41 | Weeks-months | Yes / Yes |
|  | Anhydroerythromycin | 23893-13-2 | 716 | --- | 0.435 | 3.74 | 1.41 | Weeks-months | - / - |
|  | Sulfamethoxazole | 723-46-6 | 253 | 1.9/5.7 | 3942 | 0.89 | 1.54 | Days-weeks | Yes / Yes |
|  | Trimethoprim | 738-70-5 | 290 | 7.1 | 2334 | 0.91 | 1.90 | Days-weeks | Yes / Yes |
| Anti-hypertensives | Atenolol | 29122-68-7 | 266 | 9.6 | 685 | 0.16 | 0.61 | Days | Yes / No |
|  | Irbesartan | 138402-11-6 | 429 | 4.1/4.3 ^d^ | 0.1926 | 4.81 | 3.61 | Days-weeks | Yes / No |
|  | Metoprolol | 56392-17-7 | 267 | 9.6 | 4777 | 1.88 | 1.47 | Days-weeks | Yes / No |
|  | Valsartan | 137862-53-4 | 436 | 4.0/4.6 | 1.41 | 3.65 | 2.20 | Days | Yes / No |
| Antidepressants | O-desmethylvenlafaxine | 93413-62-8 | 263 | 9.4/10.6 ^d^ | 3939 | 2.68 | 2.10 | Days-weeks | Yes / No |
|  | Venlafaxine | 99300-78-4 | 277 | 9.5 | 4371 | 0.43 | 0.74 | Days-weeks | Yes / No |
| Antiepileptics | Carbamazepine | 298-46-4 | 236 | 13.9 | 17.7 | 2.45 | 2.23 | Days-weeks | Yes / No |
| Analgesics | Acetaminophen | 103-90-2 | 151 | 9.4 | 30350 | 0.46 | 1.32 | Days | Yes / Yes |
| Antiinflamatories | Ibuprofen | 15687-27-1 | 206 | 5.3 | 41.0 | 3.97 | 2.35 | Days | Yes / No |
|  | Ketoprofen | 22071-15-4 | 254 | 4.0 | 120 | 3.12 | 2.08 | Days | Yes / Yes |
|  | Naproxen | 22204-53-1 | 230 | 4.2 | 145 | 3.18 | 1.97 | Days | Yes / No |
| Lipids regulators | Atorvastatin | 134523-03-8 | 559 | 4.3/14.9 ^d^ | 0.001 | 6.36 | 2.60 | Days | Yes / No |
|  | Gemfibrozil | 25812-30-0 | 250 | 4.8 | 4.96 | 4.77 | 2.85 | Days-weeks | Yes / No |
| Fungicides | Clotrimazole | 23593-75-1 | 345 | 4.1 | 0.030 | 6.26 | 4.79 | Weeks | Yes / No |
|  | Fluconazole | 86386-73-4 | 306 | 2.3 | 13660 | 0.5 | 0.86 | Days-weeks | Yes / No |
|  | Miconazole | 22916-47-8 | 416 | --- | 0.011 | 6.25 | 4.83 | Weeks-months | Yes / Yes ^c^ |
| Antihelmintics | Thiabendazole | 148-79-8 | 201 | 4.6 | 335 | 2.47 | 2.69 | Days-weeks | Yes / Yes |

^a^ US EPA; Estimation Program Interface (EPI) Suite. Ver. 4.1; ^b^ European Medicines Agency <https://www.ema.europa.eu/en/medicines/field_ema_web_categories%253Aname_field/Veterinary?search_api_views_fulltext=carbamazepine>; ^c^ Miconazole nitrate;

^d^ Pubchem

**Table S2**. Details of the questionnaires completed to collect information related to building characteristics, inhabitants habits and PhACs consumption.

**Building characteristics**

Type of building (flat/detached house)

Building edification age (year)

Children (number)

Teenagers (number)

Adults (number)

Do you leave your outdoor shoes at the front door?

Has the kitchen been vacuumed?

Sofas, armchairs vacuumed apart from the floor?

Furniture or other surfaces/materials vacuumed apart from the floor?

Number of times the house/office/classroom is vacuumed/washed per month (n)

Natural ventilation (min/week)

**Surroundings**

Location type (city/countryside)

Surroundings of the location house/office/school (industrial site/agricultural site/residential zone/other)

Proximity to a hospital (< 500 m)?(no/sí/centro salud)

Has your house/office/classroom been treated with products against moulds/fungus, or algae/green deposits?

**Have used in the last year? (yes/no)**

Antidepressants (venlafaxine and o-desmethylvenlafaxine)

Antiepileptics (carbamazepine)

Antibiotics (azithromycin, clarithromycin, erythromycin, sulfamethoxazole and trimethoprim)

Antyhypertensives (atenolol, metorpolol, irbesartan and valsartan)

Lipid regulators (atorvastatin and gemfibrozil)

Analgesics (acetaminophen/paracetamol)

Anti-inflammatories (ibuprofen, ketoprofen and naproxen)

Antifungal (clotrimazole, fluconazole and miconazole)

Antihelmintics (thiabendazole)


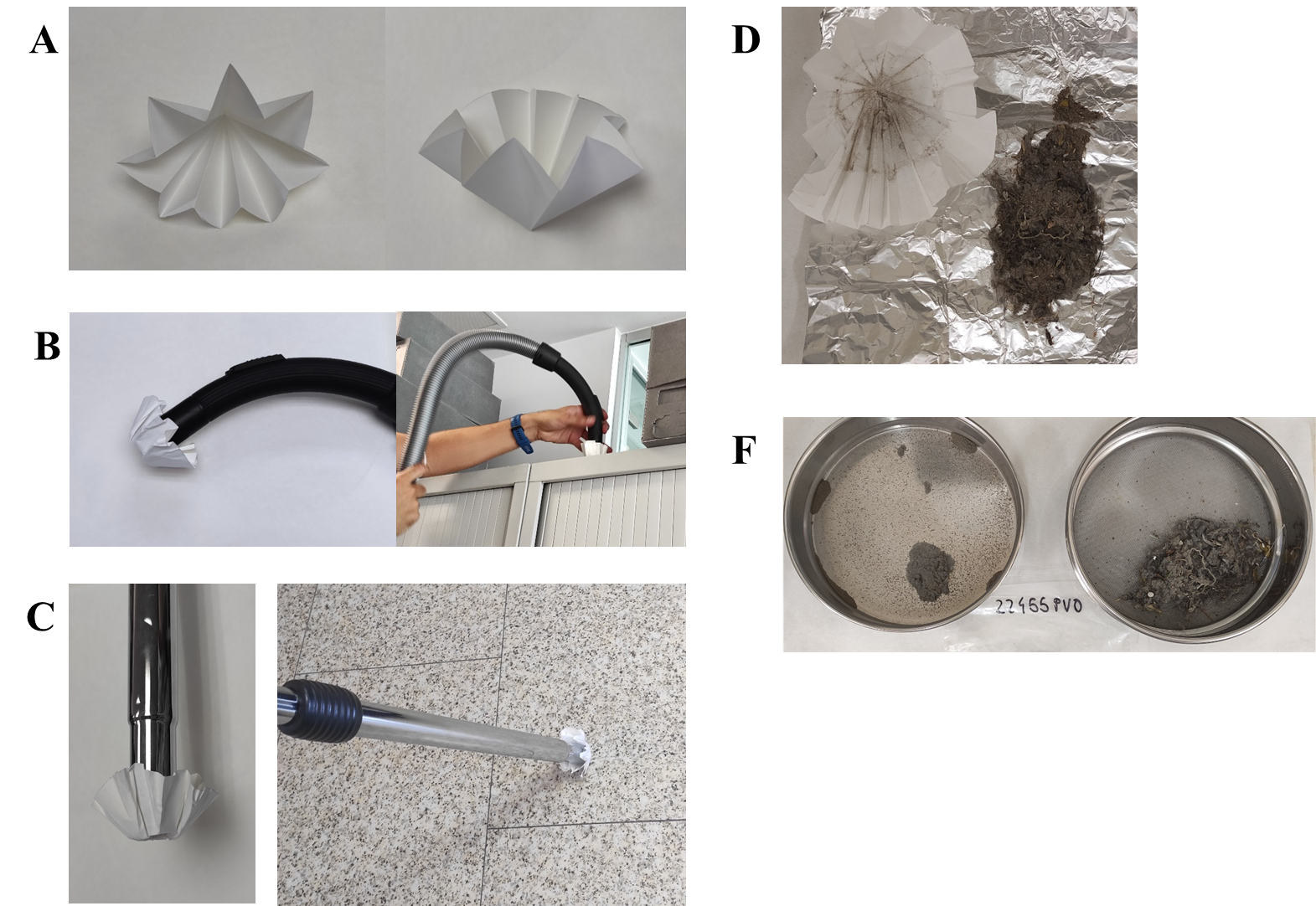


**Figure S1.** (A) Paper filter used for offices and classrooms sampling. (B) Vacuum cleaner arrangement for suspended (>0.5m) dust sampling. (C) Vacuum cleaner arrangement for deposited (floor) dust sampling. (D) Dust sample collected. (F) Sieved (500 µm) dust sample.

**S2. Instrumental analysis.**

**Table S3.** UHPLC-ESI conditions.

| **Chromatographic conditions** | | | |
| --- | --- | --- | --- |
| **Column** | Luna Omega C18 100Å column (100 mm x 2.1 mm i.d 1.6 µm.particle size) (Phenomenex) | | |
| **Column temperature** | 40ºC | | |
| **Injection volume** | 5 µL | | |
| **Mobile phase** | A: Formic acid 0.02% | | |
|  | B: MeOH | | |
| **Gradient** | Min | % A | % B |
|  | 0 | 95 | 5 |
|  | 1 | 95 | 5 |
|  | 8.5 | 0 | 100 |
|  | 14 | 0 | 100 |
|  | 14.1 | 95 | 5 |
|  | 19 | 95 | 5 |
| **Flow rate** | 0.3500 mL/min | | |
| **Triple Quad parameters** | | | |
| Ionization mode | Positive and negative | | |
| ESI temperature | 550 ºC | | |
| Curtain gas pressure | 25 psi | | |
| Ion source Gas 1 and 2 | 60 psi | | |
| Ion Spray Voltage | 5500 V | | |
| Collision Gas | 7 | | |
| Entrance Potential | 10 V | | |

The target compounds were determined in multiple reaction monitoring (MRM) mode acquiring two transitions for each target analyte (Table S4). The transitions between the precursor ion and the two most abundant fragment ions (SRM1 and SRM2) were used for quantification and identification purposes (Table S4). For deuterated standards, only one transition (quantification) was used.

**Table S4.** Precursors and product ions (quantifier and qualifier), collision energies (CE), declustering potential (DP) and collision cell exit potential (CXP) selected for the Multiple Reaction Mode (MRM) analysis.

| **PhACs** | **SRM 1 Quantifying transition (m/z)** | **DP/CE/CXP**  **(V)** | **SRM 2 Qualifying transition (m/z)** | **DP/CE/CXP**  **(V)** |  |  |
| --- | --- | --- | --- | --- | --- | --- |
| **PI Mode** |  |  |  |  |  | |
| Acetaminophen | 152 🡪 110 | 40/20/10 | 152 🡪 65 | 40/40/10 |  | |
| Acetaminophen-d3 | 155 🡪 111 | 40/20/10 |  |  |  | |
| Atenolol | 267.1 🡪 145 | 100/35/8 | 1267.1 🡪 90 | 100/25/8 |  | |
| Atenolol-d7 | 274 🡪 145 | 60/35/8 |  |  |  | |
| Atorvastatin | 559.3 🡪 250 | 80/40/10 | 559.3 🡪 292 | 80/30/10 |  | |
| Azithromycin | 749.5 🡪 158 | 160/47/8 | 749.5 🡪 83 | 160/47/8 |  | |
| Carbamazepine | 237.1 🡪 194.1 | 90/35/10 | 237.1 🡪 193.1 | 90/45/12 |  | |
| Clarithromycin | 748.5 🡪 158 | 110/33/10 | 748.5 🡪 590.4 | 110/27/16 |  | |
| Clotrimazole | 277.1 🡪 165.1 | 111/31/6 | 345.1 🡪 277.1 | 1/15/8 |  | |
| Erythromycin | 734.6 🡪 158 | 90/37/15 | 734.6 🡪 83 | 90/40/15 |  | |
| Anhydroerythromycin | 716.4 🡪 158 | 80/45/12 | 716.4 🡪 558.2 | 80/21/18 |  | |
| Fluconazole | 307.1 🡪 220 | 70/27/12 | 307.1 🡪 238 | 70/23/8 |  | |
| Irbesartan | 429.2 🡪 207.1 | 90/31/6 | 429.2 🡪 180.1 | 90/57/8 |  | |
| Ketoprofen | 255 🡪 105 | 130/33/12 | 255 🡪 209 | 130/12/12 |  | |
| Metoprolol | 268.1 🡪 116.1 | 60/25/12 | 268.1 🡪 133 | 60/35/10 |  | |
| Miconazole | 415.1 🡪 159 | 60/41/10 | 415.1 🡪 123 | 60/91/14 |  | |
| Naproxen | 231 🡪 185 | 56/21/14 | 231 🡪 170 | 56/37/10 |  | |
| O-desmethylvenlafaxine | 264 🡪 58 | 46/51/26 | 264 🡪 107 | 46/39/12 |  | |
| Sulfamethoxazole | 254.1 🡪 156 | 61/23/12 | 254.1 🡪 92 | 61/39/6 |  | |
| Sulfamethoxazole-d4 | 258 🡪 160 | 61/23/12 |  |  |  | |
| Thiabendazole | 202 🡪 175 | 91/25/12 | 202 🡪 131 | 91/55/8 |  | |
| Trimethoprim | 291.2 🡪 261.2 | 86/35/14 | 291.2 🡪 230.1 | 86/33/18 |  | |
| Valsartan | 436.2 🡪 235.2 | 70/25/10 | 436.2 🡪 291.1 | 70/25/36 |  | |
| Venlafaxine | 278.1 🡪 58 | 46/57/8 | 278.1 🡪 260 | 46/17/10 |  | |
| Venlafaxine-d6 | 284.1 🡪 64 | 46/57/8 |  |  |  | |
| **NI Mode** |  |  |  |  |  | |
| Gemfibrozil | 249.2 🡪 121.1 | -65/-22/-7 | 249.2 🡪 127.1 | -65/-14/-9 |  | |
| Gemfibrozil-d6 | 255.2 🡪 121.1 | -65/-22/-7 |  |  |  | |
| Ibuprofen | 205 🡪 159 | -20/-10/-10 | 205 🡪 161 | -20/-10/-17 |  | |
| Ibuprofen-d3 | 208 🡪 162 | -20/-10/-17 |  |  |  | |

**S3. Analytical method validation**

The analytical method was validated according to SANTE/2020/12830 (European Commission, 2020) and SANTE/12682/2019 (European Commission, 2019) guidance documents for independent laboratory validation. Validation was carried out with a household dust sample collected in a previous study (PhAC concentrations below limits of detection (LODs) in all cases) that was fortified at two concentration levels (5 and 50 ng/g). Limits of quantification (LOQs), corresponding to the lowest validated level fulfilled criteria for recovery (70 - 120%), precision (relative standard deviation ≤ 20%), and identification (MS/MS ion ratio within 30%), are shown in Table S5. LODs were calculated as the concentration giving a signal to noise ratio of 3:1 from the qualifying transition of the lowest validated level (LOQ).

**Table S5.** Limits of detection (LODs), limits of quantification (LOQs) and validation results.

|  |  |  | **Low validation level** | | | **High validation level** | | | |
| --- | --- | --- | --- | --- | --- | --- | --- | --- | --- |
| **PhAC** | **LOD**  **(ng/g)** | **LOQ**  **(ng/g)** | **Concentration**  **(ng/g)** | **Recovery**  **(%)** | **RSD**  **(%)** | **Concentration**  **(ng/g)** | **Recovery**  **(%)** | **RSD**  **(%)** |  |
| Acetaminophen | 16 | 50 | 5 | 25 | 16 | 50 | 82 | 9 |  |
| Atenolol | 3 | 5 | 5 | 94 | 14 | 50 | 84 | 15 |  |
| Atorvastatin | 7 | 50 | 5 | 16 | 11 | 50 | 74 | 18 |  |
| Azithromycin | 3 | 5 | 5 | 94 | 9 | 50 | 100 | 8 |  |
| Carbamazepine | 2 | 5 | 5 | 94 | 13 | 50 | 90 | 5 |  |
| Clarithromycin | 2 | 5 | 5 | 107 | 19 | 50 | 102 | 17 |  |
| Clotrimazole | 2 | 5 | 5 | 98 | 19 | 50 | 91 | 7 |  |
| Erythromycin | 2 | 5 | 5 | 86 | 19 | 50 | 78 | 12 |  |
| Anhydroerythromycin | 3 | 5 | 5 | 82 | 15 | 50 | 87 | 11 |  |
| Fluconazole | 2 | 5 | 5 | 82 | 16 | 50 | 87 | 15 |  |
| Gemfibrozil | 3 | 5 | 5 | 88 | 14 | 50 | 94 | 11 |  |
| Ibuprofen | - | - | 5 | 14 | 12 | 50 | 25 | 13 |  |
| Irbesartan | 1 | 5 | 5 | 77 | 13 | 50 | 93 | 12 |  |
| Ketoprofen | - | - | 5 | 22 | 20 | 50 | 35 | 18 |  |
| Metoprolol | 3 | 5 | 5 | 87 | 16 | 50 | 83 | 16 |  |
| Miconazole | 3 | 5 | 5 | 89 | 15 | 50 | 96 | 8 |  |
| Naproxen | 3 | 5 | 5 | 116 | 5 | 50 | 97 | 7 |  |
| O-desmethylvenlafaxine | 1 | 5 | 5 | 97 | 16 | 50 | 94 | 16 |  |
| Sulfamethoxazole | 3 | 5 | 5 | 90 | 10 | 50 | 86 | 18 |  |
| Thiabendazole | 3 | 5 | 5 | 92 | 11 | 50 | 86 | 12 |  |
| Trimethoprim | 1 | 5 | 5 | 83 | 14 | 50 | 83 | 9 |  |
| Valsartan | 3 | 5 | 5 | 96 | 16 | 50 | 81 | 12 |  |
| Venlafaxine | 1 | 5 | 5 | 107 | 8 | 50 | 97 | 11 |  |

Ibuprofen and ketoprofen did not fulfil SANTE guidance criteria.

**S4. Human exposure assessment**

**Table S6.** Parameter used for EDIs calculation.

|  | **Central case scenario** | | | **Worst case scenario** | | |  |
| --- | --- | --- | --- | --- | --- | --- | --- |
|  | **Toddlers** | **Adolescents** | **Adults** | **Toddlers** | **Adolescents** | **Adults** | **Ref** |
| **IR inhalation (m3/day)** | 8.9 | 15.2 | 16 | 14 | 22 | 21 | USEPA 2011 (page 14) |
| **F home** | 0.8 | 0.7 | 0.7 | 0.8 | 0.9 | 0.7 | USEPA 2011 (page 1155) |
| **F school** | 0.2 | 0.3 | 0.2 | 0.2 | 0.4 | 0.2 | USEPA 2011 (page 1160) |
| **F office** | 0.02 | 0.1 | 0.3 | 0.02 | 0.4 | 0.3 | USEPA 2011 (page 1161) |
| **BW (kg)** | 14 | 57 | 80 | 14 | 57 | 80 | USEPA 2011 (page 481) |
| **PEF (m3/g)** | 1.36E+06 | 1.36E+06 | 1.36E+06 | 1.36E+06 | 1.36E+06 | 1.36E+06 | Iwegbue et al., 2019 |
| **IngR (g/day)** | 50 | 20 | 20 | 100 | 20 | 60 | De la Torre et al., 2020 |
| **DAS (mg/cm2)** | 0.04 | 0.01 | 0.01 | 0.04 | 0.01 | 0.01 | De la Torre et al., 2020 |
| **ESA (cm2/day)** | 2564 | 4615 | 4615 | 2564 | 4615 | 4615 | De la Torre et al., 2020 |
| **AFdermal** | 0.5 | 0.5 | 0.5 | 1.0 | 1.0 | 1.0 | OECD, 2019 |
| **EF home (day/year)^a^** | 365 | 365 | 365 | 365 | 365 | 365 |  |
| **EF school (day/year)** | 175 | 175 | 175 | 175 | 175 | 175 | Ministry of Education, Vocational  Training and Sport,2023 |
| **EF office (day/year)^b^** | 271 | 271 | 271 | 271 | 271 | 271 |  |
| **ED ( year)** | 3.0 | 14 | 35 | 3.0 | 14 | 35 | Duong et al., 2023 |
| **AT (day)** | 25550 | 25550 | 25550 | 25550 | 25550 | 25550 | Duong et al., 2023 |

**^a^:** exposure frequency calculated for the total number of days a person spends at home. **^b^:** exposure frequency calculated for the total number of days a person spends in working environments, considering 42 days of holidays and 52 weekends.

**Table S7**. Quantification frequencies (Qf %), median (min. – max.) concentrations (ng/g) obtained in dust from homes, classrooms and offices.

| **Group** | **PhAC** | **Homes (n=14)** | | **Classrooms (n=48)** | | **Offices (n=23)** | | **Total (n=85)** | |
| --- | --- | --- | --- | --- | --- | --- | --- | --- | --- |
| Antidepressants | O-Desmethylvenlafaxine | 0% | - | 0% | - | 0% | - | 0% | - |
|  | Venlafaxine | 0% | - | 0% | - | 0% | - | 0% | - |
| Antiepileptics | Carbamazepine | 3/14 (21%) | 66 (27 - 130) | 1/48 (2%) | 103 | 3/23 (13%) | 51 (45 - 478) | 7/85 (8%) | 66 (27 - 478) |
| Antibiotics | Azithromycin | 0% | - | 0% | - | 0% | - | 0% | - |
|  | Clarithromycin | 0% | - | 0% | - | 1/23 (4%) | 13 | 1/85 (1%) | 13 |
|  | Erythromycin | 2/14 (14%) | 61 (39 - 84) | 0% | - | 0% | - | 2/85 (2%) | 61 (39 - 84) |
|  | Anhydroerythromycin | 0% | - | 14/48 (29%) | 15 (5.1 - 34) | 2/23 (9%) | 8.7 (7.2 - 10) | 16/85 (19%) | 14 (5.1 - 34) |
|  | Sulfamethoxazole | 1/14 (7%) | 10 | 4/48 (8%) | 26 (5.2 - 37) | 2/23 (9%) | 21 (17 - 26) | 7/85 (8%) | 19 (5.2 - 37) |
|  | Trimethoprim | 0% | - | 3/48 (6%) | 15 (6.5 - 39) | 0% | - | 3/85 (4%) | 15 (6.5 - 39) |
| Antihypertensives | Atenolol | 2/14 (14%) | 115 (39 – 190) | 0% | - | 0% | - | 2/85 (2%) | 115 (39 – 190) |
|  | Irbesartan | 2/14 (14%) | 17 (12 - 22) | 3/48 (6%) | 8.4 (7.0 – 32) | 2/23 (9%) | 10 (5.6- 14) | 7/85 (8%) | 12 (5.6 - 32) |
|  | Metoprolol | 1/14 (7%) | 351 | 0% | - | 0% | - | 1/85 (1%) | 351 |
|  | Valsartan | 1/14 (7%) | 93 | 0% | - | 0% | - | 1/85 (1%) | 93 |
| Lipid regulators | Atorvastatin | 0% | - | 0% | - | 0% | - | 0% | - |
|  | Gemfibrozil | 0% | - | 0% | - | 0% | - | 0% | - |
| Analgesics | Acetaminophen | 14 / 14 (100%) | 678 (209 - 18048) | 38/48 (79%) | 121 (51 - 1427) | 20/23 (87%) | 104 (52- 2702) | 72/85 (85%) | 166 (51 - 18048) |
| Anti-inflammatories | Ibuprofen* | 0% | - | 0% | - | 0% | - | 0% | - |
|  | Ketoprofen* | 0% | - | 0% | - | 0% | - | 0% | - |
|  | Naproxen | 0% | - | 1/48 (2%) | 630 | 1/23 (4%) | 37 | 2/85 (2%) | 334 (37 – 630) |
| Antifungal | Clotrimazole | 10/14 (71%) | 22 (5.3 - 904) | 33/48 (69%) | 28 (7.9 - 148) | 7/23 (30%) | 15 (7.0 - 36) | 50/85 (59%) | 25 (5.3 - 904) |
|  | Fluconazole | 1/14 (7%) | 143 | 1/48 (2%) | 141 | 5/23 (22%) | 22 (18 - 486) | 7/85 (8%) | 73 (18 - 486) |
|  | Miconazole | 2/14 (14%) | 10 (8.7 - 11) | 5/48 (10%) | 6.9 (5.5 - 16) | 2/23 (9%) | 116 (24 - 208) | 9/85 (11%) | 11 (5.5 - 208) |
| Antihelmintics | Thiabendazole | 12/14 (86%) | 72 (14 - 333) | 42/48 (88%) | 77 (6.3 - 431) | 13/23 (57%) | 73 (5 - 1960) | 67/85 (79%) | 74 (5 - 1960) |

*: did not fulfil SANTE guidance criteria.

**Table S8**. Quantification frequencies (Qf %) and median (min. – max.) concentration (ng/g) obtained in dust from kindergarten and high school classrooms.

| **Group** | **PhAC** | **Kindergarten**  **classrooms**  **(n=21)** | | | **High School**  **classrooms**  **(n=27)** | | |
| --- | --- | --- | --- | --- | --- | --- | --- |
| Antidepressants | O-Desmethylvenlafaxine | 0% | - | 0% | | - |  |
|  | Venlafaxine | 0% | - | 0% | | - |  |
| Antiepileptics | Carbamazepine | 1/21 (5%) | 103 | 0% | | - |  |
| Antibiotics | Azithromycin | 0% | - | 0% | | - |  |
|  | Clarithromycin | 0% | - | 0% | | - |  |
|  | Erythromycin | 0% | - | 0% | | - |  |
|  | Anhydroerythromycin | 0% | - | 14/27 (52%) | | 15 (5.1 - 34) |  |
|  | Sulfamethoxazole | 3/21 (14%) | 19 (5.2 - 37) | 1/27 (4%) | | 33 |  |
|  | Trimethoprim | 1/21 (5%) | 39 | 2/27 (7%) | | 11 (6.5 – 15) |  |
| Antihypertensives | Atenolol | 0% | - | 0% | | - |  |
|  | Irbesartan | 1/21 (5%) | 8.4 | 2/27 (7%) | | 19 (7.0 – 32) |  |
|  | Metoprolol | 0% | - | 0% | | - |  |
|  | Valsartan | 0% | - | 0% | | - |  |
| Lipid regulators | Atorvastatin | 0% | - | 0% | | - |  |
|  | Gemfibrozil | 0% | - | 0% | | - |  |
| Analgesics | Acetaminophen | 13/21 (62%) | 79 (57 - 697) | 25/27 (93%) | | 223 (51- 1427) |  |
| Anti-inflammatories | Ibuprofen | 0% | - | 0% | | - |  |
|  | Ketoprofen | 0% | - | 0% | | - |  |
|  | Naproxen | 0% | - | 1/27 (4%) | | 630 |  |
| Antifungal | Clotrimazole | 13/21 (62%) | 25 (12 - 115) | 20/27 (74%) | | 29 (7.9 - 148) |  |
|  | Fluconazole | 0% | - | 1/27 (4%) | | 141 |  |
|  | Miconazole | 4/21 (19%) | 6.9 (5.5 – 16) | 1/27 (4%) | | 15 |  |
| Antihelmintics | Thiabendazole | 21/21 (100%) | 79 (6.3 - 429) | 21/27 (78%) | | 72 (20 - 431) |  |

**
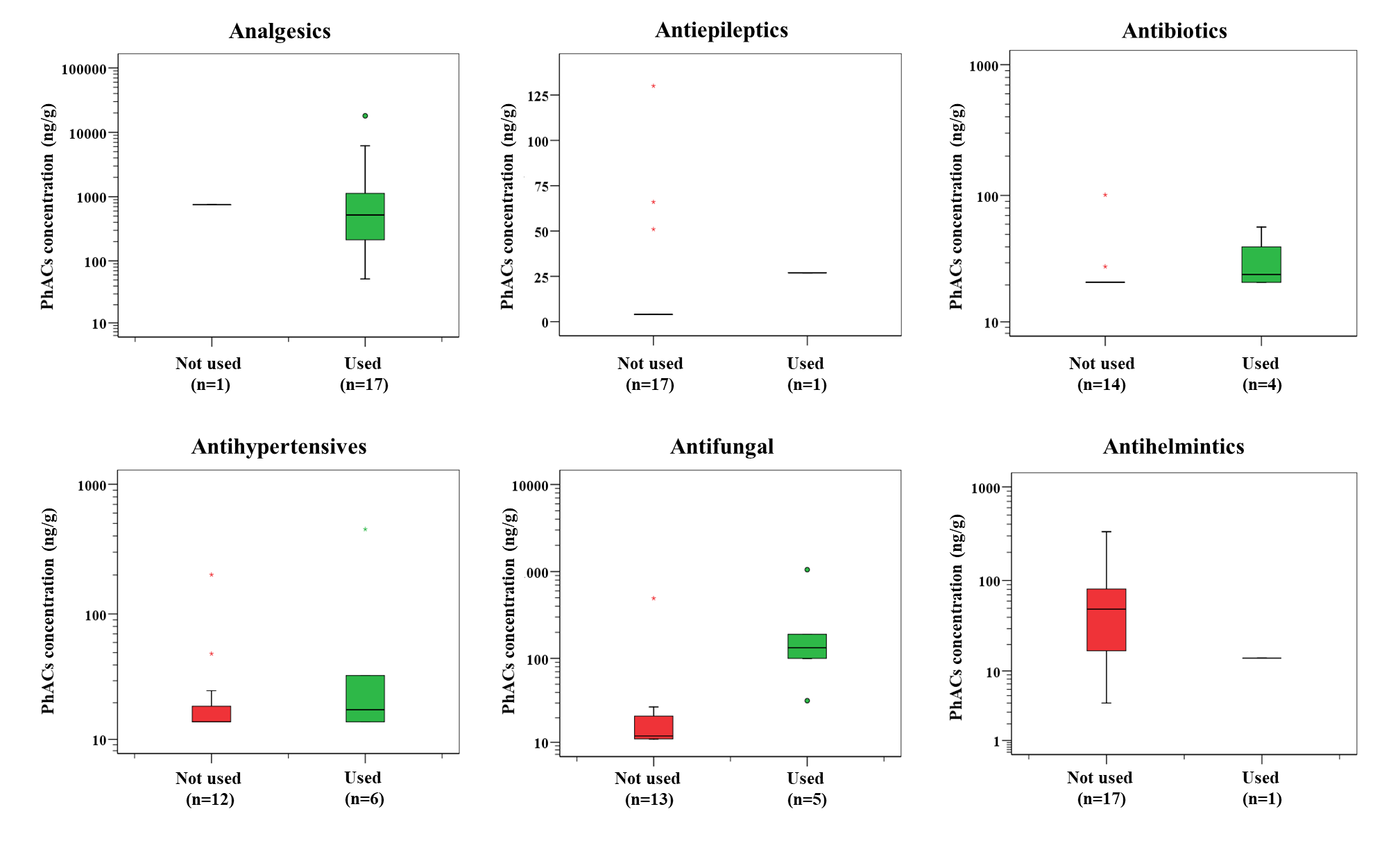
**

**Figure S2.** Box and whiskers plot of the pharmaceutical concentration by therapeutic groups related to usage information gathered from the questionnaires.

**Table S10.** Estimated daily intakes (ng/kg bw/day) via dust inhalation (EDI inhalation), ingestion (EDI ingestion), and dermal absorption (EDI dermal) calculated for toddlers, adolescents and adults at central (P50) and worst (P95) scenarios. Only PhACs that were quantified in at least two samples were considered for EDIs estimation.


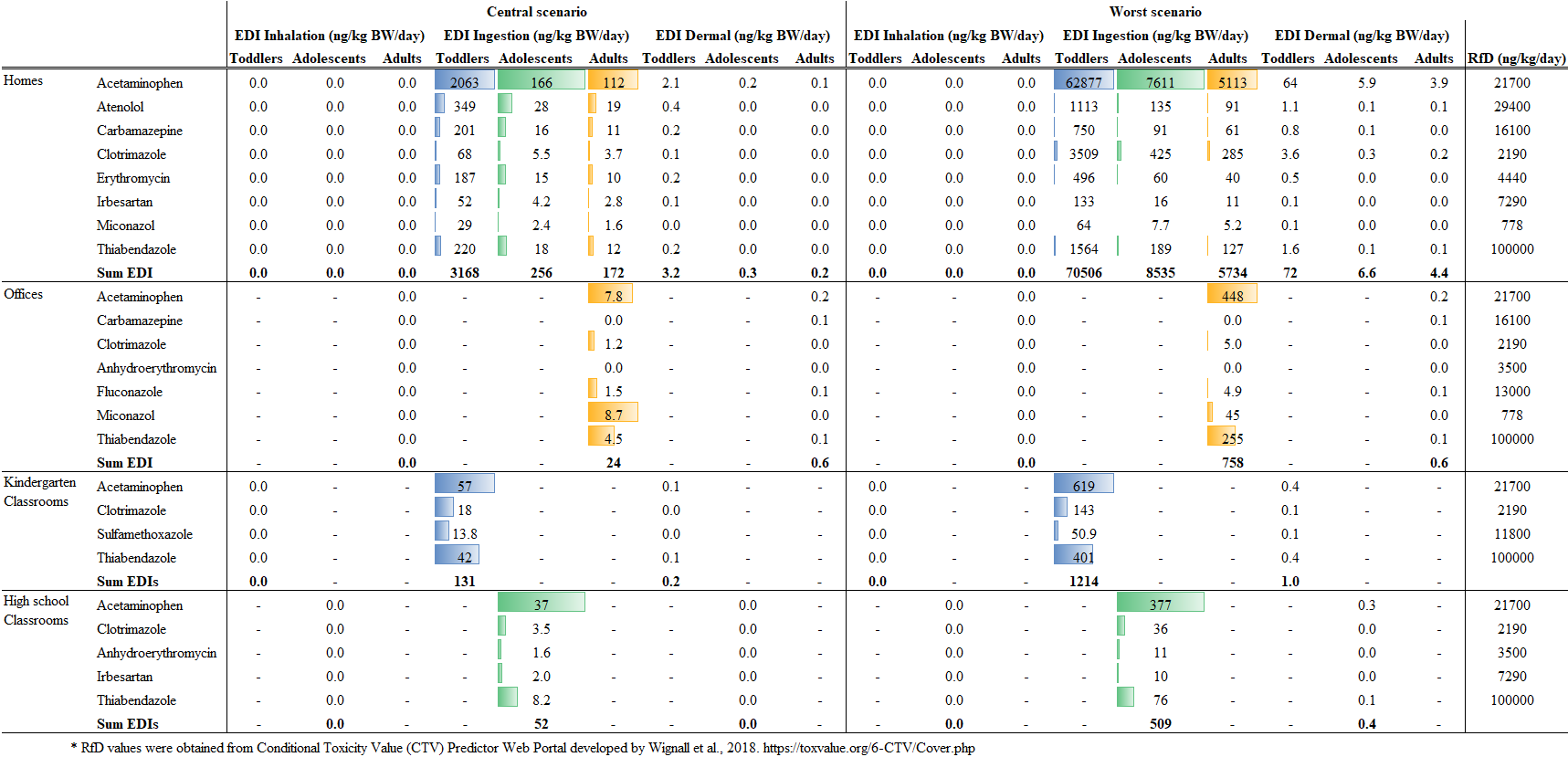


**Table S11.** Hazard Quotient (HQ) calculated for toddlers, adolescents, and adults at central (P50) and worst (P95) scenarios. (T: toddlers; Ad: adolescents, A: adults).

|  |  | **Central scenario** | | | | | | | | | | **Worst scenario** | | | | | | | | | |
| --- | --- | --- | --- | --- | --- | --- | --- | --- | --- | --- | --- | --- | --- | --- | --- | --- | --- | --- | --- | --- | --- |
|  |  | **HQ (%)**  **(EDI inhalation)** | | | | **HQ (%)**  **(EDI ingestion)** | | | **HQ (%)**  **(EDI dermal)** | | | | **HQ (%)**  **(EDI inhalation)** | | | **HQ (%)**  **(EDI ingestion)** | | | **HQ (%)**  **(EDI dermal)** | | |
|  |  | **T** | **Ad** | **A** | **T** | | **Ad** | **A** | **T** | **Ad** | **A** | **T** | | **Ad** | **A** | **T** | **Ad** | **A** | **T** | **Ad** | **A** |
| House | Acetaminophen | <0.01 | <0.01 | <0.01 | 0.41 | | 0.15 | 0.03 | <0.01 | <0.01 | <0.01 | <0.01 | | <0.01 | <0.01 | 12.4 | 7.02 | 11.8 | 0.01 | 0.01 | <0.01 |
|  | Atenolol | <0.01 | <0.01 | <0.01 | 0.05 | | 0.02 | 0.03 | <0.01 | <0.01 | <0.01 | <0.01 | | <0.01 | <0.01 | 0.16 | 0.09 | 0.15 | <0.01 | <0.01 | <0.01 |
|  | Carbamazepine | <0.01 | <0.01 | <0.01 | 0.05 | | 0.02 | 0.03 | <0.01 | <0.01 | <0.01 | <0.01 | | <0.01 | <0.01 | 0.20 | 0.11 | 0.19 | <0.01 | <0.01 | <0.01 |
|  | Clotrimazole | <0.01 | <0.01 | <0.01 | 0.13 | | 0.05 | 0.08 | <0.01 | <0.01 | <0.01 | <0.01 | | <0.01 | <0.01 | 6.87 | 3.88 | 6.51 | 0.01 | <0.01 | <0.01 |
|  | Erythromycin | <0.01 | <0.01 | <0.01 | 0.18 | | 0.07 | 0.11 | <0.01 | <0.01 | <0.01 | <0.01 | | <0.01 | <0.01 | 0.48 | 0.27 | 0.45 | <0.01 | <0.01 | <0.01 |
|  | Irbesartan | <0.01 | <0.01 | <0.01 | 0.03 | | 0.01 | 0.02 | <0.01 | <0.01 | <0.01 | <0.01 | | <0.01 | <0.01 | 0.08 | 0.04 | 0.07 | <0.01 | <0.01 | <0.01 |
|  | Miconazole | <0.01 | <0.01 | <0.01 | 0.16 | | 0.06 | 0.10 | <0.01 | <0.01 | <0.01 | <0.01 | | <0.01 | <0.01 | 0.35 | 0.20 | 0.33 | <0.01 | <0.01 | <0.01 |
|  | Thiabendazole | <0.01 | <0.01 | <0.01 | 0.01 | | <0.01 | 0.01 | <0.01 | <0.01 | <0.01 | <0.01 | | <0.01 | <0.01 | 0.07 | 0.04 | 0.06 | <0.01 | <0.01 | <0.01 |
|  | **∑HQs** | **<0.01** | **<0.01** | **<0.01** | **1.03** | | **0.39** | **0.65** | **<0.01** | **<0.01** | **<0.01** | **<0.01** | | **<0.01** | **<0.01** | **20.6** | **11.6** | **19.6** | **0.02** | **0.01** | **<0.01** |
| Office | Acetaminophen | - | - | <0.01 | - | | - | 0.01 | - | - | <0.01 | - | | - | <0.01 | - | - | 0.77 | - | - | <0.01 |
|  | Carbamazepine | - | - | <0.01 | - | | - | <0.01 | - | - | <0.01 | - | | - | <0.01 | - | - | <0.01 | - | - | <0.01 |
|  | Clotrimazole | - | - | <0.01 | - | | - | 0.02 | - | - | <0.01 | - | | - | <0.01 | - | - | 0.09 | - | - | <0.01 |
|  | Anhydroerythromycin | - | - | <0.01 | - | | - | <0.01 | - | - | <0.01 | - | | - | <0.01 | - | - | <0.01 | - | - | <0.01 |
|  | Fluconazole | - | - | <0.01 | - | | - | <0.01 | - | - | <0.01 | - | | - | <0.01 | - | - | 0.01 | - | - | <0.01 |
|  | Miconazole | - | - | <0.01 | - | | - | 0.42 | - | - | <0.01 | - | | - | <0.01 | - | - | 2.14 | - | - | <0.01 |
|  | Thiabendazole | - | - | <0.01 | - | | - | <0.01 | - | - | <0.01 | - | | - | <0.01 | - | - | 0.09 | - | - | <0.01 |
|  | **∑HQs** | **-** | **-** | **<0.01** | **-** | | **-** | **0.46** | **-** | **-** | **<0.01** | **-** | | **-** | **<0.01** | **-** | **-** | **3.10** | **-** | **-** | **<0.01** |
| Kindergarten Classrooms | Acetaminophen | <0.01 | - | - | 0.01 | | - | - | <0.01 | - | - | <0.01 | | - | - | 0.06 | - | - | <0.01 | - | - |
|  | Clotrimazole | <0.01 | - | - | 0.02 | | - | - | <0.01 | - | - | <0.01 | | - | - | 0.13 | - | - | <0.01 | - | - |
|  | Sulfamethoxazole | <0.01 | - | - | <0.01 | | - | - | <0.01 | - | - | <0.01 | | - | - | 0.01 | - | - | <0.01 | - | - |
|  | Thiabendazole | <0.01 | - | - | <0.01 | | - | - | <0.01 | - | - | <0.01 | | - | - | 0.01 | - | - | <0.01 | - | - |
|  | **∑HQs** | **<0.01** | **-** | **-** | **0.03** | | - | - | **<0.01** | - | - | **<0.01** | | - | - | **0.21** | - | - | **<0.00** | - | - |
| High school Classrooms | Acetaminophen | - | <0.01 | - | - | | 0.02 | - | - | <0.01 | - | - | | <0.01 | - | - | 0.17 | - | - | <0.01 | - |
|  | Clotrimazole | - | <0.01 | - | - | | 0.02 | - | - | <0.01 | - | - | | <0.01 | - | - | 0.16 | - | - | <0.01 | - |
|  | Anhydroerythromycin | - | <0.01 | - | - | | <0.01 | - | - | <0.01 | - | - | | <0.01 | - | - | 0.03 | - | - | <0.01 | - |
|  | Irbesartan | - | <0.01 | - | - | | <0.01 | - | - | <0.01 | - | - | | <0.01 | - | - | 0.01 | - | - | <0.01 | - |
|  | Thiabendazole | - | <0.01 | - | - | | <0.01 | - | - | <0.01 | - | - | | <0.01 | - | - | 0.01 | - | - | <0.01 | - |
|  | **∑HQs** | **-** | **<0.01** | **-** | **-** | | **0.04** | **-** | **-** | **<0.01** | **-** | - | | **<0.01** | - | - | **0.37** | - | - | **<0.01** | - |

**Table S12.** Hazard Index (HI) calculated for toddlers, adolescents, and adults at central (P50) and worst (P95) scenarios.

|  |  | **Central scenario** | | | **Worst scenario** | | |
| --- | --- | --- | --- | --- | --- | --- | --- |
|  |  | **HI (%)** | | | **HI (%)** | | |
|  |  | **Toddlers** | **Adolescents** | **Adults** | **Toddlers** | **Adolescents** | **Adults** |
| House | Acetaminophen | 0.41 | 0.15 | 0.26 | **12.4** | **7.02** | **11.8** |
|  | Atenolol | 0.05 | 0.02 | 0.03 | 0.16 | 0.09 | 0.15 |
|  | Carbamazepine | 0.05 | 0.02 | 0.03 | 0.20 | 0.11 | 0.19 |
|  | Clotrimazole | 0.13 | 0.05 | 0.08 | **6.87** | **3.88** | **6.52** |
|  | Erythromycin | 0.18 | 0.07 | 0.11 | 0.48 | 0.27 | 0.45 |
|  | Irbesartan | 0.03 | 0.01 | 0.02 | 0.08 | 0.04 | 0.07 |
|  | Miconazole | 0.16 | 0.06 | 0.10 | 0.35 | 0.20 | 0.33 |
|  | Thiabendazole | 0.01 | <0.01 | 0.01 | 0.07 | 0.04 | 0.06 |
|  | **∑HQs** | **1.03** | **0.39** | **0.65** | **20.6** | **11.7** | **19.6** |
| Office | Acetaminophen | - | - | 0.01 | - | - | 0.77 |
|  | Carbamazepine | - | - | <0.01 | - | - | <0.01 |
|  | Clotrimazole | - | - | 0.02 | - | - | 0.09 |
|  | Anhydroerythromycin | - | - | <0.01 | - | - | <0.01 |
|  | Fluconazole | - | - | <0.01 | - | - | 0.01 |
|  | Miconazole | - | - | 0.42 | - | - | **2.14** |
|  | Thiabendazole | - | - | <0.01 | - | - | 0.09 |
|  | **∑HQs** | **-** | **-** | **0.46** | **-** | **-** | **3.10** |
| Kindergarten Classrooms | Acetaminophen | 0.01 | - | - | 0.06 | - | - |
|  | Clotrimazole | 0.02 | - | - | 0.13 | - | - |
|  | Sulfamethoxazole | <0.01 | - | - | 0.01 | - | - |
|  | Thiabendazole | <0.01 | - | - | 0.01 | - | - |
|  | **∑HQs** | **0.03** | **-** | **-** | **0.21** | **-** | **-** |
| High school Classrooms | Acetaminophen | **-** | 0.02 | **-** | **-** | 0.17 | - |
|  | Clotrimazole | **-** | 0.02 | **-** | **-** | 0.16 | - |
|  | Anhydroerythromycin | **-** | <0.01 | **-** | **-** | 0.03 | - |
|  | Irbesartan | **-** | <0.01 | **-** | **-** | 0.01 | - |
|  | Thiabendazole | **-** | <0.01 | **-** | **-** | 0.01 | - |
|  | **∑HQs** | **-** | **0.04** | **-** | **-** | **0.37** | **-** |

**REFERENCES**

De la Torre, A., Navarro, I., Sanz, P., Martinez, M. A., 2020. Organophosphate compounds, polybrominated diphenyl ethers and novel brominated flame retardants in European indoor dust: Use, evidence for replacements and assessment of human exposure. J. Hazard. Mater., 382, 121009. <https://doi.org/10.1016/j.jhazmat.2019.121009>

Duong, H. T., Kadokami, K., Nguyen, D. T., Trinh, H. T., Doan, N. H., Mizukawa, H., Takahashi, S., 2023. Occurrence, potential sources, and risk assessment of pharmaceuticals and personal care products in atmospheric particulate matter in Hanoi, Vietnam, Environ. Sci. Pollut. Res., 30, 34814-34826. <https://doi.org/10.1007/s11356-022-24630-0>

European Commission, 2019. Analytical Quality Control and Method Validation Procedures for Pesticide Residues Analysis in Food & Feed. SANTE/ 12682/2019. Available online at https://www.eurl-pesticides.eu/userfiles/file/EurlALL/AqcGuidance_SANTE_2019_12682.pdf

European Commission, 2020. Pesticide Analytical Methods for Risk Assessment and Post-approval Control and Monitoring Purposes. SANTE/2020/12830 Rev.1. Available online at https://food.ec.europa.eu/system/files/2021-03/pesticides_ppp_app-proc_guide_res_mrl-guidelines-2020-12830.pdf

Iwegbue, C. M. A., Obi, G., Uzoekwe, S. A., Egobueze, F. E., Odali, E. W., Tesi, G. O., Nwajei, G. E., Martincigh, B. S., 2019. Distribution, sources and risk of exposure to polycyclic aromatic hydrocarbons in indoor dusts from electronic repair workshops in southern Nigeria. Emerging Contaminants, 5, 23-30. <https://doi.org/10.1016/j.emcon.2018.12.003>

Ministry of Education, Vocational Training and Sport. Education system in Spain. https://www.educacionyfp.gob.es/atencion-educativa-ucranianos/sistemas-educativos (accessed 22 December 2023).

OECD, 2019. Guidance notes on dermal absorption. Environment, Health and Safety Publications Series on Testing and Assessment, nº 156 (Draft Second Edition). Available at <https://www.oecd.org/chemicalsafety/testing/Guidance%20Notes%20Dermal%20Absorption%20156_Oct2019_clean.pdf>

Royano, S., De la Torre, A., Navarro, I., Martinez, M. A., 2023. Pharmaceutically active compounds (PhACs) in surface water: Occurrence, trends and risk assessment in the Tagus River Basin (Spain). Sci. Total. Environ., 905, 167422. <https://doi.org/10.1016/j.scitotenv.2023.167422>

United States Environmental Protection Agency (U.S. EPA), 2011. Update for Chapter 5 of the Exposure Factors Handbook - Soil and Dust Ingestion. EPA/600/R-17/384F. <https://cfpub.epa.gov/ncea/risk/recordisplay.cfm?deid=236252>

Wignall, J. A., Muratov, E., Sedykh, A., Guyton, K. Z., Tropsha, A., Rusyn, I., Chiu, W. A., 2018. Conditional Toxicity Value (CTV) Predictor: An In Silico approach for generating quantitative risk estimates for chemicals. Environ. Health Perspect., 126 (5), 057008. [https://doi.org/10.1289/EHP29](https://doi.org/10.1289/EHP2998)98
